# Cerebrovascular Insult as Presenting Symptom of Neurofibromatosis Type 2 in Children, Adolescents, and Young Adults

**DOI:** 10.3389/fneur.2018.00733

**Published:** 2018-09-10

**Authors:** Isabel Gugel, Victor-Felix Mautner, Lan Kluwe, Marcos Soares Tatagiba, Martin Ulrich Schuhmann

**Affiliations:** ^1^Department of Neurosurgery, University Hospital Tübingen, Tübingen, Germany; ^2^Centre of Neurofibromatosis and Rare Diseases, University Hospital Tübingen, Tübingen, Germany; ^3^Department of Neurology, University Medical Center Hamburg-Eppendorf, Hamburg, Germany; ^4^Department of Maxillofacial Surgery, University Medical Center Hamburg-Eppendorf, Hamburg, Germany; ^5^Division of Pediatric Neurosurgery, University Hospital Tübingen, Tübingen, Germany

**Keywords:** neurofibromatosis type 2, stroke, vasculopathy, aneurysm, presenting symptom

## Abstract

**Background and Purpose:** Neurofibromatosis Type 2 (NF2) is an autosomal-dominant tumor-prone disorder characterized by the manifestations of central nervous system lesions. However, the first clinical signs of disease are often non-tumorous. Cerebrovascular insults are known in NF2, however, not yet described as first symptom in young NF2 patients.

**Methods:** Magnetic resonance image scans of 298 NF2 patients treated in our neurofibromatosis center in Tübingen from 2003 to 2017 were retrospectively evaluated focusing on presence of aneurysms and ischemic stroke. Clinical data were used to clarify whether or not ischemic stroke or aneurysm rupture were the first presentation of disease. Blood of the patients were subjected to genetic screening for constitutional *NF2* mutations.

**Results:** We identified 5 cases under age of 25 years with aneurysms or ischemic stroke. Among them three had ischemic strokes of the brain stem and one aneurysmal subarachnoid hemorrhage as the first symptom of the disease. Incidental finding of 2 intracranial aneurysm occurred in one patient. All aneurysms were clipped. Patients with ischemia suffered from dysarthria, gait disturbances, dizziness, and hemiparesis. Residual signs of hemiparesis and dysarthria persisted in one patient. All others fully recovered from the cerebrovascular insult. Bilateral vestibular schwannomas and intracranial meningiomas were found in all five patients.

**Conclusions:** A cerebrovascular insult in the vertebrobasilar territory may occur as first symptom of disease in young NF2 patients. The brain stem seems to be especially prone to ischemic stroke. Multicenter studies on large NF2 cohorts are needed to determine the prevalence and pattern of cerebrovascular insults and disease in NF2 patients.

## Introduction

Neurofibromatosis type 2 (NF2) is an autosomal-dominant inherited tumor-prone disorder with an incidence of 1:27.000 (1). The genetic cause is the heterozygotic inactivation of the NF2 tumor suppressor gene on Chromosome 22q12p, encoding for the protein merlin (moesin-ezrin-radixin-like protein). Truncating NF2 mutations (e.g., nonsense and frameshift mutations) are more frequently associated with severe phenotype, whereas non-truncating mutations such as missense mutations often lead to the milder phenotypes (2–5). Splicing NF2 mutations are reported to be associated with various phenotypes (5–7).

Apart from the typical occurrence of bilateral vestibular schwannomas (VS) patients suffer from other central and peripheral nervous system tumors such as meningiomas, schwannomas, and ependymomas. Patients are also characterized by abnormalities of the skin (e.g., subcutaneous schwannomas), eye (e.g., juvenile cataract, retinal hamartoma), and bony changes (e.g., scoliosis) (8). In childhood and adolescence early manifestation like neuropathy, eye axis deviation, or scoliosis occur long before the typical hearing impairment and provide additional opportunities for early diagnosis (9–11).

Cerebrovascular disease with ischemic events or aneurysms has been reported mostly in single cases, and most of the time not as presenting symptom.

Herein, we report ischemic brain stem stroke in 3 young NF2 patients aged from 7 to 22 years, as the first symptom of the disease. Furthermore, one 17-year-old patient presented with aneurysmal subarachnoid hemorrhage (aSAH) as initial manifestation of NF2. One 16-year-old patient harbored two unruptured aneurysms of the middle cerebral artery (MCA).

## Materials and methods

All NF2 patients meeting the NIH criteria and treated at the Department of Neurosurgery Tübingen and Centre of Neurofibromatosis Tübingen from 2003 to 2017 were retrospectively evaluated. Informed consent was obtained from all patients. Use of samples and clinical data for this study were approved by the Ethics Board of the Medical Faculty and University Hospital of Tübingen.

Thin-slice magnetic resonance imaging (MRI) of the brain with and without contrast agent was assessed during routine diagnostics for intracranial NF2 manifestations. Clinical notes and MRI scans and reports were retrospectively evaluated focusing on ischemic stroke and presence of intracranial aneurysms. Most of those routine MRI scans did not contain specific magnetic resonance angiography (MRA) sequences, which would be much better suited to discover incidental aneurysms, thus the thinnest available T2-weighted axial and coronal scans were used in absence of MRA to screen for intracranial aneurysms.

Patients with cerebrovascular disease underwent MRA of the head and neck. In 3 of 5 cases digital subtraction cerebral angiography (DSA) was performed. Magnetic resonance spectroscopy (MRS) was completed in one case to exclude suspected glioma. Stroke patients underwent a detailed cardiac (e.g., electrocardiogramm, transesophageal echocardiography), laboratory (standard and extended coagulation tests) and cervical vascular (doppler) work-up to exclude extracranial reasons for stroke as part of the routine work-up in stroke.

All patients were subjected for mutation analysis for the *NF2* gene using DNA from blood as previously described (12).

## Results

**Figure 1:**
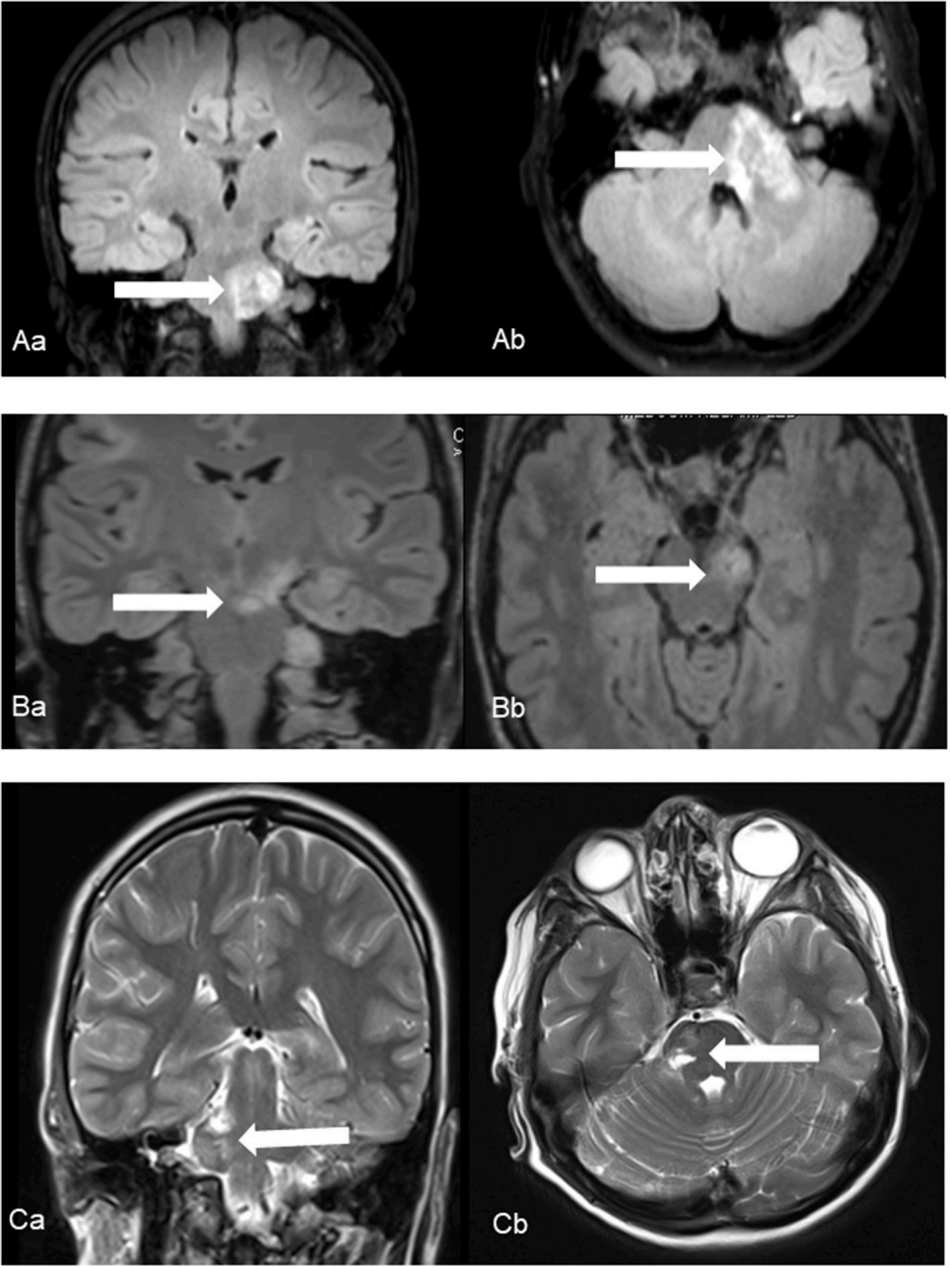
Ischemic events in patients No. 1 **(A)**, No. 2 **(B)** and No. 3 **(C)** using coronal **(a)** and axial **(b)** fluid-attenuated inversion recover (Patient No. 1 and 2) and T2-weighted (Patient No. 3) images. Characteristic foci of increased T2 intensity **(arrows)** are identified in the left ponto-medullary transition/cerebellar peduncle (Patient No. 1), in the left cerebral peduncle (Patient No. 2) and in the right pons (Patient No. 3).

**Figure 2:**
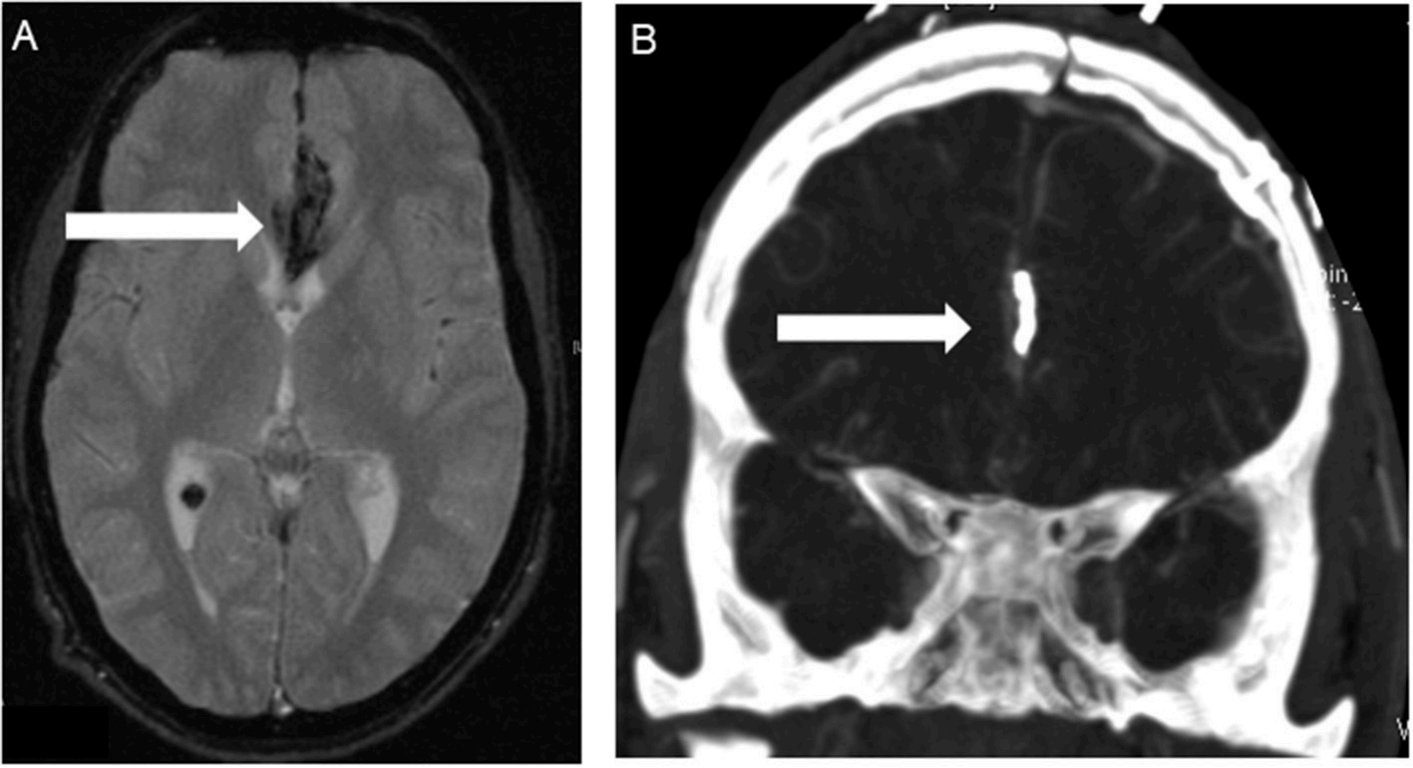
Patient No. 5 with subarachnoid hemorrhage because of ruptured left pericallosal artery aneurysm. **(A)** T2-wighted MRI scans showed SAH in the frontal interhemispheric region **(arrow)** due to the pericallosal artery aneurysm. **(B)** Postoperative computed tomography angiography (CTA) confirmed correct positioning of the clip **(arrow)** without any findings of vasospasm or ischemia.

**Figure 3:**
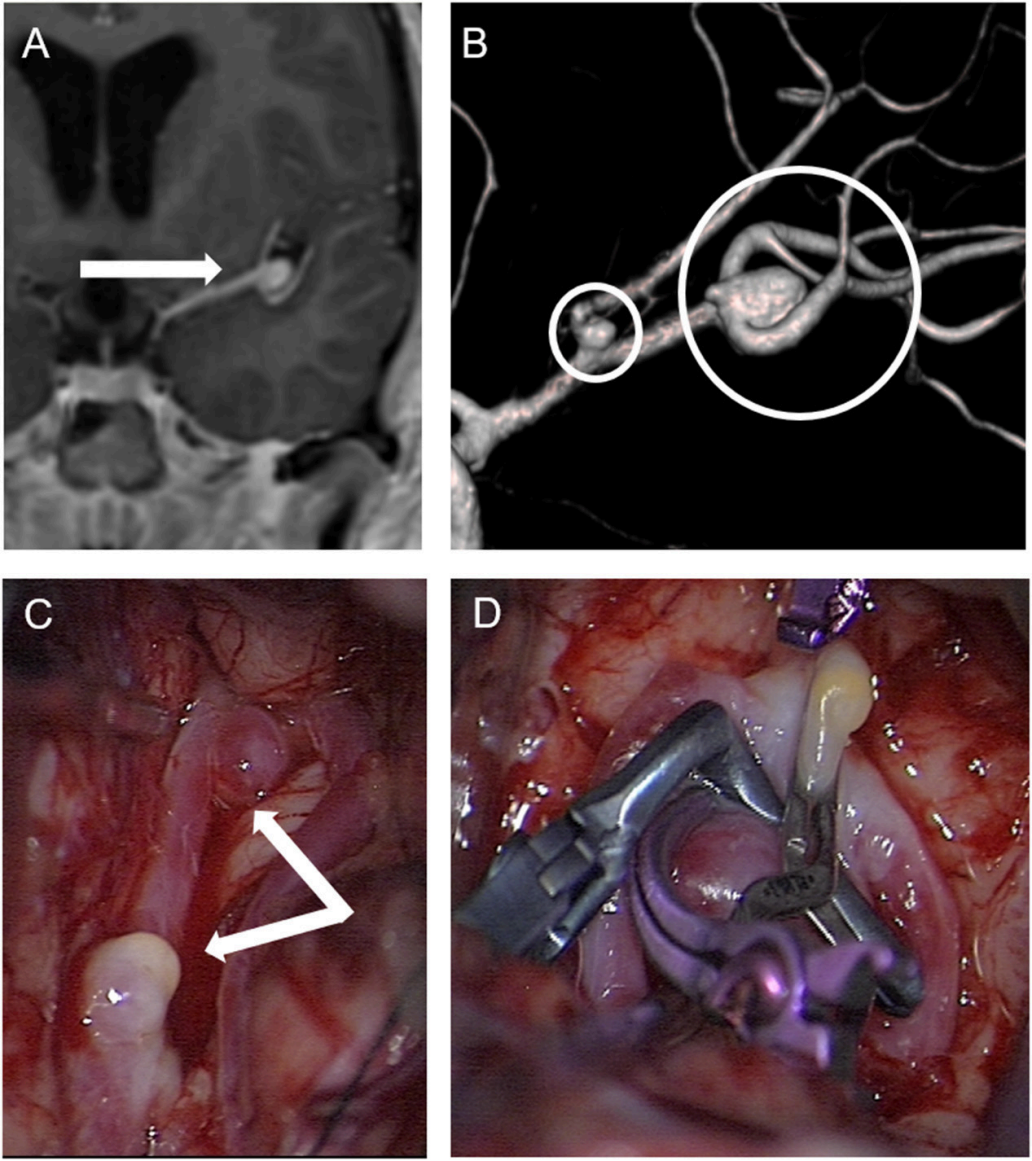
Patient No. 4 with incidental finding of two aneurysms in the left middle cerebral artery. **(A)** Enhanced coronal T1-weighted MRI for planning vestibular schwannoma surgery demonstrated an aneurysm in the middle cerebral artery bifurcation (arrow) and completion of cerebral angiography and three-dimensional computed angiography **(B)** detected another aneurysm originating from the frontal branch of the middle cerebral artery (circles) in patient No. 4. **(C)** Preparation of both aneurysms in the sylvian fissure of the left side via a pterional approach. and **(D)** microsurgical clipping.

A total of 298 NF2 (mean age at time of diagnosis: 17.83 ± 9.20 years, range: 1–35 years; 45.3% male, 54.7% females) cases were included in primary analysis. Age cut-off for inclusion in this study was 25 years at onset of event. Based on history and MRI scans, cerebrovascular disease was found in 5 cases. Ischemic stroke of the brain stem was detected as presenting symptomatology in three patients at an age of 7, 13, and 22 years (Figure 1). In one 17-year-old boy aSAH occurred as first symptom (Figure 2), while in a 16-year-old girl, newly diagnosed for NF2 due to hearing impairment, two incidental MCA aneurysms were found (Figure 3).

For detailed patient characteristics see Table 1. None of the patients was previously diagnosed for NF2, none of them had any cardiovascular risk factors and cardiac, vascular and laboratory diagnostic was inconspicuous for extracranial stroke reasons. Conventional MRI with contrast agent revealed the presence of bilateral vestibular schwannoma (VS) and intracranial meningiomas in all five patients. Emergent MRA confirmed the above-mentioned findings as well as DSA, which was completed for all aneurysms. Open biopsy was performed in one ischemic patient for further analysis since a glioma was suspected. Histopathology showed no signs of neoplasm or vasculitis, but reactive astrogliosis, and activation of microglia as well as a high presence of erythrocytes. But despite the detected hyalinized and large-caliber vessels, generalized vasculopathy was excluded by DSA and MRA.

**Table 1:** Characteristics of reported NF2 patients with cerebrovascular manifestations.

**Patient No**.	**Age at time of diagnosis (years)**	**Cerebral vasculopathy**	**Clinical presentation**	**Clinical outcome (3 months)**	**Diagnostic work-up**	**Therapy**	**Genetics**	**Other NF2 manifestations**
1	13	Ischemic stroke of left ponto-medullary transition/cerebellar peduncle	Dizziness, gait & taste disturbances, left-sided hypacusis, hemiparesis and facial palsy H&B II, dysarthria, ataxia	Complete recovery regarding stroke symptoms	MRI, MRA, MRS, DSA, open biopsy	Aspirin	c.447+1G>A, splicing mutation	Bilateral VS, intracranial meningiomas, spinal ependymomas, scoliosis, juvenile cataract, left ocular motility disorder
2	22	Ischemic stroke of left cerebral peduncle	Hemiparesis and facial palsy, dysarthria, dizziness, fine motor skill disorders	Residual hemiparesis and mild dysarthria	MRI, MRA, MRS	No	c.115-1G>C, splicing mutation	Bilateral VS, intracranial meningiomas, spinal ependymomas and schwannomas
3	7	Ischemic stroke in the pons of the right side	Hemiparesis, dysarthria	Complete recovery regarding stroke symptoms	MRI, MRA, MRS	No	c.114 G>A, last position of exon 1, putative splicing mutation	Bilateral VS, intracranial meningiomas, spinal ependymomas, meningiomas, PNP, retinal hamartoma
4	16	Two aneurysms of the left MCA	Asymptomatic regarding aneurysm symptoms	–	MRI, MRA, DSA	Clipping	c.1122+1G>T, splicing mutation	Bilateral VS, intracranial meningiomas, spinal ependymomas, peripheral schwannomas
5	17	One aneurysm of the left pericallosal artery	SAH bleeding with intracranial pressure symptoms	Hemihypesthesia, cognitive disorders	MRI, MRA, DSA	Clipping	c.178_179insTGAC p.Trp60 Leufs^*^27, frameshift mutation	Bilateral VS, intracranial meningiomas, spinal ependymomas

*MRS, Magnetic Resonance Spectroscopy; DSA, Digital Subtraction Angiography; MRA, Magnetic Resonance Angiography; H&B, House and Brackmann Classification System (13). All patients have a negative family history regarding NF2*.

Patients with brainstem ischemia were all symptomatic for the cerebrovascular insult. The strokes occurred in left ponto-medullary transition/cerebellar peduncle, in the left cerebral peduncle and in the right pons, respectively. All three patients showed a hemiparesis of the contralateral side and dysarthria, two of them suffered from a facial palsy and dizziness. Fine motor skill disorders occurred in one patient. Retrospectively, one patient had beginning symptoms of bilateral vestibular schwannomas such as hypacusis and taste disturbances prior to stroke, the others had no prior symptoms related to the presence of VS.

Over a period of 6 months, two ischemic stroke patients did fully recover from their ischemia associated symptoms. Mild residual hemiparesis and dysarthria persisted in one patient. All patients underwent surgical treatment of their VS in the further course at a time of beginning hearing impairment.

Spontaneous subarachnoid hemorrhage (SAH) from a left pericallosal artery aneurysm occurred in a 17-year-old boy prior to diagnosis of NF2 (Figure 2). The aneurysm was clipped and the patient fully recovered from the event. Later on, he underwent treatment of his newly discovered vestibular schwannomas.

A 16-year-old girl presenting with very large vestibular schwannomas with massive brain stem compression was diagnosed of NF2 due to beginning hearing loss. Routine MRI showed in addition a suspicion of a left MCA aneurysms. This was further investigated by MRA and DSA, revealing two aneurysms of the left MCA. Both aneurysms were clipped prior to surgical treatment of the large VS (Figure 3).

Mutations in the *NF2* gene were found in all patients. Three of the mutations (patient 1, 2, and 4) were in typical splicing sites and they therefore are expected to alter the *NF2* splicing products. In the third patient the mutation was at the last position of the exon which is not a typical splicing site. However, this position may also affect the spicing process and the mutation can be defined as putative splicing mutation. The last patient (5) carried a truncating frameshift mutation. No patients exhibited a family history for NF2.

## Discussion

To the best of our knowledge only 8 further cases of cerebral aneurysms in NF2 patients have been reported in literature (see Table 2). In 7/8 the diagnosis of NF2 was known. In 5 of those 8 patients, all adults aged over 30 years, incidental aneurysms were described. 4 of those 5 come from one UK series with 104 NF2 patients (14), which also contained one additional case of SAH due to aneurysm rupture. This resulted in a prevalence of 4.4% for aneurysms in this NF2 patients series of the UK, slightly higher than the 2–3% in the general population (21).

**Table 2:** Intracranial cerebrovascular disease and insults reported in literature in NF2 patients.

**Study**	**Age**	**Symptoms**	**Imaging findings**	**Residual clinical findings**	**Vascular risk factors**	**NF2 mutation**	**First symptom of NF2**
Afridi et al. (14)^*1^	40	Subarachnoid hemorrhage	MCA aneurysm	NA ^*4^	HTN, Smoker	Frameshift mutation in exon 5	No
Afridi et al. (14)^*1^	35	Asymptomatic	Ophthalmic ICA aneurysm	No finding	No	Whole gene deletion	No
Afridi et al. (14)^*1^	35	Asymptomatic	MCA and ICA aneurysm clip	No finding	HTN, Ex-Smoker	Not detected	No
Afridi et al. (14)^*1^	31	Asymptomatic	MCA bifurcation aneurysm	No finding	No	Nonsense mutation in exon 15	No
Afridi et al. (14)^*1^	48	Asymptomatic	Cavernous ICA aneurysm	NA ^*4^	No	Frameshift mutation in exon 1	No
Alanin et al. (15) ^*3^	23	Epileptic seizures	Ruptured mycotic meningeal media aneurysm	Increased intracranial pressure and death	Bevacizumab	NA ^*4^	No
Singla et al. (16)^*2^	23	NA ^*4^	Ruptured posterior cerebral artery aneurysm	NA ^*4^	NA ^*4^	NA ^*4^	NA ^*4^
Lesley et al. (17)^*2^	36	Asymptomatic, detected during workup	Unrupted aneurysma of the middle meningeal artery	Asymptomatic	No	NA ^*4^	No
Sreedher et al. (18)^*2^	2	Ataxia	Stroke in the left brachium pontis	Not described	No	c.169 C>T, nonsense mutation in exon 2	Yes
Ng et al. (19)^*2^	15	Acute right-sided weakness, right facial palsy, mild tongue deviation to the right	Large left-sided infarction from the midbrain to the pons	Hemiparesis	No	c.448–1G>A, splicing mutation in exon 5	No
Ryan et al. (20)^*2^	18	Acute dysphasia, right hemiparesis, right homonymous hemianopsia, right facial weakness	Infarction in the left MCA territory, irregularity of the left MCA, stenosed insular branch	Moderate recovery of right upper and lower limb function, aphasia persisted	No	c.600-2A>G, splicing mutation in exon 7	No

*ICA, Internal Carotid Artery; HTN, Hypertension; NA, Not available*.

*1 *Retrospective analysis of 114 NF2 cases in adults. 5/114 cases showed intracranial aneurysm*.

*2 *Case reports*.

*3 *Incidental finding in a retrospective analysis investigating the effect of bevacizumab on NF2-associated vestibular schwannomas*.

*4 *Information was not available*.

In our German series based on standard MRI scans without a specific angiography protocol, 2/298 patients harbored intracranial aneurysms, resulting in a prevalence of 0.7%. This retrospective analysis on the basis of routine MRI scans with a suspected lower sensitivity than MRA thus most likely underestimates the true prevalence of intracranial aneurysms in NF2 patients. Furthermore, both of our patients with aneurysms were adolescents and not adults. The incidence in this highly selected single center cohort is clearly below the incidence of the general population in Western Europe (21). Thus, the non-representative composition of both cohorts regarding the incidence of intracranial aneurysms in NF2 does not allow for any conclusions on the true incidence of intracranial aneurysms in NF2 patients.

In addition, two more aneurysmal SAH cases in NF2 were reported in single case reports, both ruptured in 23-year-old patients. Thus 3 of 4 cases of ruptured intracranial aneurysms in NF2 patients (three from the literature and one in our series), occurred in patients below the age of 25 years. The remaining patient was 40 years of age. This fact of a younger age at rupture is of note and can be interpreted as a higher vulnerability of aneurysms in NF2 patients for rupture compared to the general population. Consequently, we would recommend prophylactic treatment for incidentally discovered and/or silent aneurysm in NF2 patients rather than observation. Furthermore, we suggest to perform once a MRA as part of their routine follow-up MRI scans in all NF2 patients at the end of adolescence to detect still silent aneurysm early on. MRA should be repeated every 5 years.

So far, three cases of an ischemic cerebrovascular insult in NF2 patients have been published, all as single case reports (18–20). In a 2-year-old child, the ischemic stroke was the presenting symptom as in our cases. In this child, the area of ischemic stroke was as well in the brain stem, in the left brachium pontis (18). The initiated diagnostic work-up established the diagnosis of NF2 in this child. Genetic analysis found a truncating mutation in the NF2 gene. In all ischemic stroke cases in our study two of the mutations are typical splicing mutations while one is a putative splicing mutation.

The remaining two case reports, described ischemic stroke in two patients aged 15 and 18 years with known NF2 (19, 20). In one the area of stroke was midbrain and pons, in the other the infarction was in the left MCA territory, where a stenosed insular MCA branch was discovered as potential reason for stroke. In those two patients as well, splicing mutations were reported.

Interestingly, in literature, the most described etiology of posteror stroke identified in children without NF2 is vertebral artery dissection (VAD) and its determinants are not well known (22, 23). Due to this fact, it is important to include the vetrebro-basilar territory in diagnostic work-up to avoid underestimation of VAD. In our stroke cases, we could not find any macroangiopathic abnormality via contrast-enhanced MRI with MRA of the head and neck.

The most sensitive imaging modality has not been established in children with posterior stroke, but MRI with MRA has its known limitations in sensitivity and specify compared to endoluminal imaging techniques [DSA, computed tomography angiography (CTA)] (22, 24, 25). For inconclusive cases, endoluminal techniques, in particular DSA, can help to eliminate uncertainties (22, 24, 25). Therefore, besides the standard diagnostic work-up and exclusion of aneurysm by MRA, we would recommend to perform contrast-enhanced MRA including the vertebrobasilar vessels in childhood or beginning adolescence. X-ray-based endoluminal imaging techniques should not routinely be performed in young NF2 patients, but considered in special cases with questionable MRA findings.

In summary, our 3 cases present the largest series of ischemic cerebrovascular events as first symptoms under the age of 22, with the three more cases described in literature. In 5 of these 6 cases the stroke occurred in the midbrain/brain stem area and no obvious reason for stroke like occlusion of a visible larger vessel, especially not in the vertebral or basilar arteries could be established. Thus, a microvascular affection is the most likely cause and the midbrain/brainstem area seems to be the predilection site for ischemic events in NF2.

Furthermore, 5 of 6 patients had splicing mutations, although no distinct genetic pattern emerges from those 5 cases. The young age from 2 to 22 years of all 6 stroke patients and the occurrence in a distinct anatomical area make a genetic influence of the NF2 mutation likely in the pathophysiology of these ischemic insults. Further genetic research on the basis of multi center cohort studies including a much larger NF2 population might yield more insight in the pathophysiology.

In conclusion, the presented data however seem to suggest, that early ischemic brain stem insults below the age of 25 can be considered as a previously not recognized feature of NF2.

## Data availability statements

All datasets analyzed for this study are included in the manuscript.

## Author contributions

IG: design, methodology, investigation, data curation, formal analysis, writing-drafting the manuscript; V-FM, LK, and MT: supervision and oversight of the study and writing-review of the manuscript; MS: design, methodology, investigation, data duration, formal analysis, writing-drafting the manuscript, supervision and oversight of the study and writing-reviewing of the manuscript.

### Conflict of interest statement

The authors declare that the research was conducted in the absence of any commercial or financial relationships that could be construed as a potential conflict of interest.
